# Students’ Motivation and Affection Profiles and Their Relation to Mathematics Achievement, Persistence, and Behaviors

**DOI:** 10.3389/fpsyg.2020.533593

**Published:** 2021-01-14

**Authors:** Feiya Xiao, Li Sun

**Affiliations:** ^1^Department of Psychology, Henan Normal University, Xinxiang, China; ^2^Department of Mathematics and Statistics, Texas Tech University, Lubbock, TX, United States

**Keywords:** math achievement, math self-concept, perceived control in math, persistence, math anxiety, LPA, math interest

## Abstract

**Objective:**

We aimed to explore profiles of subgroups of United States students based on their motivational and affective characteristics and investigate the differences in math-related behaviors, persistence, and math achievement across profiles.

**Method:**

We used 1,464 United States students (male 743 51%, female 721 49%, age 15.82 ± 0.28) from PISA 2012 United States data in our study. First, we employed latent profile analysis and secondary clustering to identify subgroups of students based on motivational (math self-concept, interest in math, perceived control, and instrumental motivation) and affective factors (math anxiety). Next, we used regression to compare differences in math behavior, persistence, and achievement among all identified subgroups.

**Results:**

We found five distinct groups of students with different patterns of motivation and affection. The subgroup of students with the lowest math anxiety and the highest motivation levels showed the highest math achievement and levels of persistence. The groups with high math interest, math self-concept, and instrumental motivation showed the most frequent math-related behaviors.

**Conclusions:**

Our findings reveal the complexity of the students’ motivational and affective profiles. Our findings are significant for teachers and educators to understand the diversity of students and provide theoretical and practical support for individualized and differentiated instruction.

## Introduction

Mathematics underperformance is a critical issue among students in the United States (Geary et al., 2012). The United States students’ average math performance in international large-scale assessment ranks behind several developed countries in Asia and Europe. Insufficient math ability limits students’ educational and career development. Students who are struggling with mathematics are more likely to drop out of school or avoid math-related careers (Rozek et al., 2015). Thus, it is important for researchers and educators to understand the factors that affect students’ math performance and maintain their aspirations in learning math, which is associated with math-related activities and persistence. Therefore, proper instruction can be provided for students based on their characteristics. In response, the literature indicates that some non-cognitive factors, like math self-concept, interest, perceived control in math, instrumental motivation to learn math, and math anxiety, play critical roles in understanding students’ math behavior, persistence, and performance in math learning (Hudson, 1999; Ma and Xu, 2004; Marsh et al., 2005; Antunes and Fontaine, 2007; Schiefele, 2009; Fernández-Villaverde et al., 2015). While math self-concept, interest, perceived control in math, instrumental motivation to learn math positively associate with frequencies of math behaviors, persistence when facing difficulties and challenges, and math achievement, math anxiety is inversely correlated with them.

This view is grounded in the expectancy-value theory of achievement motivation (EVM). EVM integrates a variety of constructs into a comprehensive model based on theories on expectancy (e.g., self-efficacy theory and control theories) and theories on reasons for engagement (e.g., intrinsic motivation theories), explaining why individuals perform better on certain tasks but poorer on others (Pintrich and Schrauben, 1992; Eccles and Wigfield, 2002; Marsh and Martin, 2011; Barron and Hulleman, 2015; Ng et al., 2016; Ramirez et al., 2016; Batchelor et al., 2019). According to EVM, one’s expectancies for success and subjective task values on tasks directly determine their persistence, performance, and task choices (Eccles and Wigfield, 2002; Barron and Hulleman, 2015; Jiang et al., 2018). Students with high levels of expectancy for success and/or perceived task values are more likely to engage in learning, even in difficult situations (Murayama et al., 2013).

Expectancies for success are broad beliefs about individuals’ abilities in a specific domain (Eccles, 1983). These beliefs that an individual has explained why he/she is willing to do challenging tasks affect his/her behaviors and choices (Eccles, 1983; Eccles and Wigfield, 2002; Wigfield and Cambria, 2010). Despite some theoretical distinctions from expectancy beliefs, empirically math self-concept and perceived control in math are close to expectancy beliefs in the math domain (Eccles and Wigfield, 2002). Math self-concept refers to the student’s self-perceived competence and self-appraisal in mathematics (Marsh et al., 2005; Antunes and Fontaine, 2007). It is a domain-specific academic self-concept, which is a component of the global self-concept that develops through interaction between one’s environment and self-attribution (Gniewosz et al., 2015; Tomasetto et al., 2015). However, math self-concept is distinguished from global self-concept or non-academic self-concept construct; it is closer to the concept, math self-efficacy, both of which are self-perceived ability beliefs related to math (Ferla et al., 2009). Theoretically, there are subtle differences between math self-concept and math self-efficacy. Self-concept in math emphasizes self-appraising on the whole math domain, whereas math self-efficacy, which refers to students’ beliefs on their ability to succeed in a specific math task, emphasizes self-perception on a specific task. However, Eccles and Wigfield (2002) pointed out that based on empirical studies, children and adolescents do not distinguish between self-efficacy and self-concept. Many studies have emphasized the strong effect of academic self-concepts on important factors that are associated with learning, such as emotion (Chen et al., 2015), interest (Marsh et al., 2005), school engagement (Raufelder et al., 2013; Frawley et al., 2014), learning outcomes (Tornare et al., 2015), and career choice (Sax et al., 2015). Relatively strong linkage has been found between self-concept and math achievement (Antunes and Fontaine, 2007; McWilliams et al., 2013; Parker et al., 2013). In one study, Marsh et al. (2006) employed data from a large German study, which includes 4,475 participants from 149 randomly selected upper secondary schools, to reveal the relationship among various dimensions of self-concept and core personality constructs. The results showed that math self-concept had strong correlations with math school grades (*r* = 0.71), math standardized achievement test scores (*r* = 0.59), and the likelihood of taking advanced math courses (*r* = 0.51). Another study investigated the influence of academic self-concept and other motivational variables on later achievement based on data from PISA 2003 and 2004, which included 6,020 German students. Using structural equation modeling controlling intelligence and prior achievement, they found that academic self-concept significantly predicted later achievement, explaining 4% of the variance (Kriegbaum et al., 2015).

Perceived control in math refers to a belief that one has the controllability of what he/she thinks is important (ability, powerful others, or luck) to succeed in math (d’Ailly, 2003). It is an individual’s perception of the causal relationship between his/her math behavior and math learning outcomes (Rotter, 1966). Perceived control is associated with individual’s learning-related behavior (Patrick et al., 1993), perseverance when facing challenging tasks (Cervone and Peake, 1986), intrinsic and extrinsic motivation (Patrick et al., 1993), and learning outcomes (Stupnisky et al., 2007). Empirical studies on perceived control and academic achievement consistently found a positive correlation between these two variables (Damon et al., 2006). For example, Stupnisky et al. (2007) found that perceived control is a powerful determinant of college students’ first-year learning achievement (β = 0.14). Murayama et al. (2013) examined the impact of motivational variables like perceived control, cognitive learning strategies, and intelligence on the long-term growth of students’ math achievement. They used data drawn from a Germany longitudinal study involving annual assessments of 3,530 students from grade 5 to grade 10. Using latent growth curve model, they found that intelligence (β = 4.72) contributed to the initial level of achievement, whereas perceived control (β = 3.78), intrinsic motivation (β = 4.64), and learning strategies (β = 4.51) predicted the growth of math achievement.

Subjective values in EVM are associated with incentives or reasons for doing activities or tasks, explaining why individuals choose to or not to do certain tasks in achievement-related situations (Eccles and Wigfield, 2002; Wigfield and Cambria, 2010). Eccles posits four types of task values: intrinsic value, utility value, attainment value, and cost. The first three values are positively associated with learning activities and achievement, while the fourth one is negatively associated with the other three. In our study, we are interested in two positive value components, interest, which is the same as intrinsic values and instrumental motivation, which is the same as utility value, and the negative value component, cost. Math interest refers to positive emotion associated with math-related activities, which may contribute to an internal drive to engage in these activities for them *per se* (Hidi, 1990; Eccles and Wigfield, 2002; Lee et al., 2014; Grigg et al., 2018). Interest can be divided into individual interest (a long-lasting preference for objects and activities) and situational interest (a temporary emotional state aroused by environmental stimuli when performing a task) (Krapp, 2002, 2005; Lohbeck et al., 2016). It includes two highly correlated but distinguished aspects, emotional and cognitive (Schiefele and Csikszentmihalyi, 1995; Lohbeck et al., 2016). The emotional aspect refers to positive feelings associating with objects or activities, typically, feelings of enjoyment and involvement (Krapp, 2005). The cognitive aspect refers to the appraisal of the value of the objects or activities (Schiefele et al., 2012). Empirically, studies have found that interest is positively associated with memory, attention (Tobias, 1994), comprehension (Hidi and Anderson, 1992), sophisticated learning strategies (Hidi, 1990), time and efforts invested in learning (Macher et al., 2012; Trautwein et al., 2015), and academic achievement and performance (Hidi, 2000; Fisher et al., 2012). A longitudinal study on 168 children from a diverse background with a mean age of 4.39 showed that prior interest was significantly predicting later math skills (β = 0.13) with control for initial skills and intervention status (Fisher et al., 2012).

Instrumental motivation refers to the external drive to involve in tasks or activities because of practical or pragmatic reasons, such as improving career prospects and opportunities (Hudson, 1999). Studies concerning instrumental motivation started from second-language learning area. Lukmani (1972) examined the association between English proficiency and different types of motivation for learning English among a group of high school students and found that instrumental motivation more strongly correlated with English proficiency than integrative motivation. The researchers recently found the important role of instrumental motivation in science learning (Yu, 2012; Acosta and Hsu, 2014; Mujtaba and Reiss, 2014; Perera, 2014). Studies have found that students with higher instrumental motivation for science are more likely to continue learning science-related subjects even when they are not compulsory and tend to have higher achievement in science. Since 2003, large-scale international assessment started to investigate instrumental motivation to learn mathematics. Based on all these, instrumental motivation might have the same effects on math learning.

As mentioned above, an abundance of studies has demonstrated linkages between ability beliefs, positive dimensions of task values, and learning. Only until recently, researchers have started to explore the negative dimension of subject task value, the cost, to fully explain the reason why students engage in learning (Jiang et al., 2018). Cost refers to a perception of the negative aspect of engaging in a task (Eccles and Wigfield, 2002; Barron and Hulleman, 2015; Jiang et al., 2018), consisting of negative emotion of anxiety and fear of failure, efforts required to succeed, and the lost opportunities to engage in another task. In our study, we focused on math anxiety, an important negative emotional aspect of cost. Math anxiety refers to unpleasant feelings like “fear, tension, and apprehension that many people experience when engaging with math” (Ashcraft, 2002; Ahmed et al., 2012) and worrying of failure when engaging in math activities (Barron and Hulleman, 2015). Math anxiety has been found negatively associated with learning and performance (Ho et al., 2000; Ma and Xu, 2004; Miller and Bichsel, 2004; Ramirez et al., 2016; Namkung et al., 2019). Ramirez et al. (2016) examined the relationship among math anxiety, math achievement, and strategy use with a sample of 564 children (256 first grader and 308 s grader). They found that math anxiety impedes advanced memory-based strategy use and impairs math achievement by diminishing the use of advanced memory-based strategies. Further, a recent twin study confirmed a negative correlation between math anxiety and performance, which is attributed to both environmental and genetic factors quantitatively (Malanchini et al., 2020).

From existing studies, we found three possible explanations of why math anxiety inversely affects persistence in learning, math behavior, and achievement. First, math anxiety causes avoidance of involving in math-related tasks and activities (Ashcraft and Faust, 1994; Chinn, 2009; Trezise and Reeve, 2014). Second, the state of anxiety interferes with working memory to a certain extent, and dysfunction of working memory causes low math achievement (Ashcraft and Kirk, 2001; Ramirez et al., 2018). Last, math anxiety may be due to a lack of mathematical ability, which may cause poor math achievement (Ramirez et al., 2018).

Theoretically, expectancy beliefs and the positive dimensions of subjective task values are positively correlated with each other (Eccles and Wigfield, 2002). Initially, in the academic domain, students’ ability beliefs and perceived task-values may be independent of each other; however, their ability beliefs may change the perception of task values gradually when they grow older (Wigfield, 1994). An early study indicated that changes in competence beliefs caused the changes in interest (Mac Iver et al., 1991). Some recent studies also showed that most students with high competency beliefs demonstrate a high perceived task value (Trautwein et al., 2012). Grigg et al. (2018) found that prior math interest positively predicted subsequent math ability beliefs (β = 0.186). On the other hand, negative emotion is negatively correlated with expectancy beliefs and task values. A prior study supported a reciprocal relationship between math self-concept and math anxiety (β = −0.07–−0.15) (Ahmed et al., 2012). The theoretical explanation is that one’s appraisal of incompetence may trigger a perceived environmental threat since one’s self-concept involves appraisal of one’s competence to cope with environmental demands (Bandura, 1997). In consequence, perceived environmental threat causes anxiety. In addition, Gottfried (1982) found that math anxiety and intrinsic motivation are negatively correlated with each other among both four graders (β = −0.54) and seventh graders (β = −0.40). The students with a high level of math interest may be more likely to accept any challenges in learning rather than avoid them, while students with high math anxiety may view challenges as threats to them, thus avoid challenging tasks.

More complex interplay of math anxiety and the motivational factors (i.e., math self-concept, perceived control, interest in math, and instrumental motivation in math) on learning has been emerging. On one hand, math anxiety negatively affects motivational factors. Math anxiety may diminish perceived values of activities or tasks, expectancy, and ultimately motivation to engage in math-related learning (Wigfield and Meece, 1988; Eccles and Wigfield, 2002; Trautwein et al., 2012). On the other hand, the motivational factors compensate for the impaired efficiency caused by math anxiety (Justicia-Galiano et al., 2017). Students with certain levels of math anxiety may not necessarily engage less in math activities if they have comparatively higher levels of motivational factors that can counteract the negative impact of math anxiety by making more efforts. With the influence of math motivation, math anxiety and math performance probably demonstrate curvilinear relation. The study of Wang et al. (2015) on 237 undergraduate students and 262 pairs of same-sex twins show the distinct patterns of the relationship between math anxiety and performance on math-related tasks at different levels of math motivation. Students with higher math motivation performed the best on math-related tasks when their math anxiety levels were at a medium level, and the worst when their math anxiety levels were extremely low or high. However, the negative linear relationship between math anxiety and math performance was found among students with lower math motivation.

Most of the past studies on math motivation and affection used variable-centered approaches and have proved the significant association between motivational and affective variables and their relations to outcome variables, like academic achievement, in an entire population or observable groups. One limitation of these studies is that they ignored the potential unobservable student subgroups based on math motivational and affective factors, which will provide nuanced information on the patterns of the levels of students’ motivation and affection related to math. To address this limitation, the person-centered approach, latent profile analysis (LPA), can be employed. LPA that can be used to identify subgroups of students based on math motivation and affection helps us to understand the characteristics of students within the group that they are assigned to in accordance with the highest probability (Spurk et al., 2020). Additionally, LPA allows us to compare differences of outcome variables between latent subgroups, thus we can understand whether the combination of the motivational and affective factors affect outcome variables, which cannot be addressed by using variable-centered approaches. To our best knowledge, there is a scant study focusing on investigating the characteristics of students’ latent profiles based on math motivational and affective variables. We only found one study (Wang et al., 2018) that showed the existence and patterns of students’ profiles based on math motivation and math anxiety. Therefore, in the current study, we aimed to identify the characteristics of students’ subgroups based on the motivational and affective factors. More specifically, we are interested to find out the different combinations of the motivational and affective factors. Although, as we mentioned before that motivational variables and math anxiety are inversely associated with each other, we did not assume that we would only find the pattern of the combination of these variables as high level of math motivation vs. low level of math anxiety or high level of math anxiety vs. low level of math motivation. The reason is that Wang et al. (2018) found eight groups of students and showed complex combinations rather than a simple “high vs. low” pattern. Moreover, we intend to examine whether there would be differences in outcome variables, i.e., math achievement, persistence, and math behaviors, between the identified students’ subgroups. The purpose of doing this is to find out which combination of motivational factors and math anxiety is most favorable for better outcomes.

## Materials and Methods

### Data

Data used in our study were derived from the Program for International Student Assessment (PISA) 2012 United States data. PISA is a large-scale international assessment that is coordinated by Organization for Economic Cooperation and Development (OECD) focusing on students’ mathematics, reading, and science competencies, as well as learning-related factors, such as self-concept, interest, anxiety, and more, approaching the end of compulsory schooling (OECD, 2014). PISA is conducted every 3 years.

### Participants

PISA 2012 includes 510,000 students from 65 countries and economies, and the major subject PISA 2012 assesses is mathematics. The participants were between the age of 15 years 3 months and age 16 years 2 months at the time of the test (OECD, 2014). More details about PISA 2012 data collection or test design can be found in the PISA 2012 Technical Report. United States data originally include a sample of 4,978 students (male = 2,525 51%, female = 2,453 49%) from 162 schools. We are using 37 response items, gender, socio-economic status, and math achievement items for this study. Of the 37 response items, 1,719 students (34.5%) miss 27 or more items, another 1,689 students (33.9%) miss 10–26 items. Among them, 1,590 students miss all items for math behavior, instrumental motivation, interest, persistence, and self-control. Another 1,540 students miss all items from anxiety and self-concept. Of all these missing data, 95.8% are due to “questionnaire rotation” by design, which complies with missing at complete random (MCAR). Thus, we used list-wise deletion, which should not bring bias to subsequent analysis under MCAR assumption (please see Supplementary Material for more details in describing missingness in this data). Finally, 1,464 participants (male 743 51%, female 721 49%, age 15.82 ± 0.28) with complete information were included in our study.

### Measures

#### Mathematics Anxiety (Anxiety)

Anxiety was measured by a five-item ANXMAT scale that was used in the main survey of PISA 2012 and PISA 2003. The items are rated on a four-point Likert scale (1 = strongly agree; 4 = strongly disagree), measuring the levels of anxiety students feel when they are involved in math-related activities (e.g., “I often worry that it will be difficult for me in mathematics classes.”) (OECD, 2014). Internal consistency in this sample was reliable (Cronbach’s α = 0.88).

#### Mathematics Self-Concept (Self-Concept)

Self-concept was measured by a five-item SCMAT scale that was used in the main survey of PISA 2012 and PISA 2003. The items are rated on a four-point Likert scale (1 = strongly agree; 4 = strongly disagree), measuring how students feel about their abilities in math (e.g., “I have always believed that mathematics is one of my best subjects.”) (OECD, 2014). Internal consistency in this sample was reliable (Cronbach’s α = 0.90).

#### Instrumental Motivation to Learn Mathematics (Instrumental Motivation)

Instrumental motivation was measured by the INSTMOT scale used in both PISA 2003 and PISA 2012, consisting of four items. The items are rated on a four-point Likert scale (1 = strongly agree; 4 = strongly disagree), measuring how much the students feel that they learn math due to the benefits it will bring for them in their future study and career (e.g., “Making an effort in mathematics is worth it because it will help me in the work that I want to do later on.”) (OECD, 2014). Internal consistency in this sample was reliable (Cronbach’s α = 0.91).

#### Mathematics Interest (Interest)

Interest was measured by four items in the main survey of PISA 2012. The items are rated on a four-point Likert scale (1 = strongly agree; 4 = strongly disagree), measuring how much the students feel they engaged in math (e.g., “I do mathematics because I enjoy it.”) (OECD, 2014). Internal consistency in this sample was reliable (Cronbach’s α = 0.92).

#### Perceived Control in Math (Perceived Control)

Perceived control was measured by six items in the main survey of PISA 2012. The items are rated on a four-point Likert scale (1 = strongly agree; 4 = strongly disagree), measuring how students feel that their success in math is due to their ability and efforts (i.e., “If I put in enough effort I can succeed in mathematics.” “Whether or not I do well in mathematics is completely up to me.” “Family demands or other problems prevent me from putting a lot of time into my mathematics work.” “If I had different teachers I would try harder in mathematics.” “If I wanted to I could do well in mathematics.” “I do badly in mathematics whether or not I study for my exams.”) (OECD, 2014). Internal consistency in this sample was not reliable (Cronbach’s α = 0.68).

#### Math-Related Behaviors (Behavior)

Behavior was measured by a newly created scale in PISA 2012. The scale consists of eight items that are rated on a four-point Likert scale (1 = always or almost always; 4 = never or rare)(OECD, 2014). This scale measures how often students were involved in math-related activities (e.g., “I talk about mathematics problems with my friends.”). Internal consistency in this sample was reliable (Cronbach’s α = 0.83).

#### Persistence in Learning Math (Persistence)

Persistence in learning math was measured by eight items that are rated on a five-point Likert scale (1 = very much like me; 5 = not at all like me). The scale measures how likely the students will persist on a task when they have difficulties (e.g., “When confronted with a problem, I do more than what is expected of me.”) (OECD, 2014). Internal consistency in this sample was not reliable (Cronbach’s α = 0.76).

#### Math Achievement (Achievement)

Achievement was measured by five plausible values based on students’ responses to math tests. Plausible values were drawn from posterior distribution estimated by item response theory, with a mean of 500 and a standard deviation of 100 (OECD, 2014). The item responses are formatted in three types: open construct-response, closed construct-response, and selected-response. Open constructed-response items are scored by trained experts based on students’ extended written responses. Closed construct-response and selected items are scored based on whether a student provides a correct answer (OECD, 2013). The PISA 2012 mathematics test items were designed within four broad areas: change and relationships, space and shape, quantity, and uncertainty and data, assessing seven fundamental mathematical capabilities: communication, mathematizing, representation, reasoning and argument, devising strategies for solving problems, using symbolic, formal, and technical language and operations, and using mathematical tools (OECD, 2013). The test items are developed based on problems that an individual may encounter in real-world settings.

#### Socio-Economic Status (SES) and Gender

It is generally accepted and proved by empirical studies that students’ family SES and gender are associated with math persistence, behavior, and achievement (Passolunghi et al., 2014). The development of expectancy and value is shaped by personal and environmental factors, like gender and SES (Eccles and Wigfield, 2002; Barron and Hulleman, 2015). Therefore, we added these two variables as covariates in our regression models (Passolunghi et al., 2014). Gender was self-reported. Students’ family information was used to produce an index to reflect SES, which is scaled to have a standard normal distribution.

### Analysis

Our data analyses were run in R (version 3.5.1) with packages reshape2 (2_1.4.3), ggplot2 (2_3.1.0), mclust (version 5.4.5), factoextra (version 1.0.5), multcomp (version 1.4–10), lavaan (version 0.6–3), lmtest (0.9–37), lsmeans (2.30), and agricolae (1.3–1).

First, arithmetic means of all response items for math anxiety, math self-concept, perceived control in math, math interest, instrumental motivation to learn math, persistence in learning math, math-related behaviors, and math achievement were calculated. For anxiety, self-concept, perceived control, interest, and instrumental motivation, the calculated means were further subtracted from 5. For persistence, the calculated mean was subtracted from 6. Thus, a larger number is corresponding to higher anxiety, perceived control, self-concept, interest, instrumental motivation, behavior, and persistence. Pairwise Pearson correlations among all eight measures were calculated. Confirmatory factor analysis (CFA) was used to confirm the validity of 24 response items measuring the five constructs (anxiety, interest, self-concept, perceived control, and instrumental motivation), which are used in later latent profile analysis (LPA) (Supplementary Table 1).

Then, LPA was employed to cluster students based on these five motivational and affective factors. Anxiety, self-concept, perceived control, interest, and instrumental motivation were further residualized using linear regressions adjusted for SES and gender to remove variations attributed to SES and gender (Hart et al., 2016). The resultant values of anxiety, self-concept, perceived control, interest, and instrumental motivation were converted to Z scores and used as input of LPA, which was done using “mclust” package in R. Model selection was conducted comprehensively considering Bayesian Information Criterion (BIC), Integrated Complete-data Likelihood (ICL), Bootstrap Likely Ratio Test (BLRT), and normalized entropy (*E*_norm_ calculated by formula 1) from 14 types of models (Scrucca et al., 2016) with 1–12 profiles. According to mclust package, these 14 types of models are defined by three-letter names, such as “EEI,” “VEI,” or “VVV.” They are basically gaussian finite mixture models with different constraints on variance–covariance structures. Scrucca (2016) has clearly explained these concepts. Briefly, the first two letters define the constraints on variances of sub-group distributions. The most restricted “EI^∗^” model means that all variables from all different groups share the same variance. The unrestricted “VV^∗^” models mean all variances from all variables of different groups are freely estimated. The third letter defines correlations among variables. “^∗∗^I” models have no correlations at all, “^∗∗^E” models have the same correlations among groups, while “^∗∗^V” models have variable correlations among groups. Normalized entropies were calculated using the following formula:

(1) $$E_{\text{norm}}=1+\frac{\sum_{i=1}^{N}\sum_{k=1}^{K}P_{\text{ik}}\,\log P_{\text{ik}}}{N\,\log K}$$

where *N* is the number of individuals, *K* is the number of groups, *P*_ik_ is the probability of individual *i* belonging to group *k*. Higher entropy means less overlapping of latent distributions, or more accurate classifications (Celeux and Soromenho, 1996). To select the model that best fits our data, we first compared BIC and ICL of all 168 models and chose several models with the best BIC and/or ICL values. Then, normalized entropies were calculated for selected models. The ones with the highest entropy were kept. Finally, if there are more than one model with the same variance–covariance structures but different number of latent groups (very likely), BLRT was conducted to test if a more complex model (with more latent groups) is significantly better than a simpler model (with less latent groups).

To increase the interpretability of our results, we conducted secondary clustering to merge groups with significant overlapping from the initial LPA output (Baudry et al., 2010). Briefly, two groups were merged at a time sequentially. Groups to be merged at each step were chosen based on a criteria that the merged clusters show the smallest entropy *E*, which is calculated using following formula:

(2) $$\text{E}=-\sum_{i=1}^{N}\sum_{k=1}^{K}P_{\text{ik}}\,\log P_{\text{ik}}$$

Thus, this method could generate results with the number of clusters from the same number of clusters from LPA down to one cluster. The number of clusters was chosen using “elbow method,” which finds the number of clusters when the decrease in entropy becomes minimum. This process was assisted by piecewise linear regression (Fraley and Raftery, 1998). The means of instrumental motivation, interest, self-concept, perceived control, and anxiety among subgroups were compared using ANOVA followed by Tukey HSD.

Last, students’ persistence, behavior (log-transformed due to right skewness), and achievements of different subgroups were compared using linear regressions with gender and SES as covariates. The effect size of subgroup membership was calculated according to Cohen (1988) using the following formula, where *R*^2^ is the differences of the *R*^2^ of the full model and the model without covariate of interest:

(3) $$f^{2}=\frac{R^{2}}{1-R^{2}}$$

The significance of variables was checked using the Likelihood Ratio Test (LRT). Significance of pairwise comparisons between all subgroups was done using the Tukey HSD *post hoc* tests.

## Results

After removing individuals with missing values in included response items, 1,464 individuals were included. Cronbach’s alpha was used to check the internal reliability of measured constructs. A value greater than 0.8 is thought as reliable (Peterson, 1994). Six constructs have values greater than 0.8, except for self-concept and perceived control, whose Cronbach’s alphas are 0.68 and 0.76, respectively (see the “Materials and Methods” section for details). Descriptive statistics of arithmetic means of response items for all the variables included in our analysis are shown in Table 1. Most of these variables are roughly symmetrically distributed. However, math behavior is right-skewed, for most students reported “sometimes” or “never or rarely” participated in math-related activities, like “talk about mathematics problems with friends.” Table 2 shows the pairwise correlations of all factors included in this study. Using Cohen’s empirical cutoffs for correlation coefficient (Cohen, 2013), interest, perceived control, self-concept, instrumental motivation, and persistence are positively correlated with each other, showing moderate (0.3 < *r* < 0.5) to strong (*r* > 0.5) correlations, except for the correlation between behavior and persistence, which showed a weak positive correlation (*r* = 0.21). Behavior correlated moderately and positively with interest and self-concept, and with instrumental motivation positively but weakly. Achievement positively and moderately correlated with perceived control, self-concept, and with persistence positively but weakly. The strongest positive correlation was observed between interest and self-concept (*r* = 0.65). Anxiety negatively correlated with all the other variables (*r* ≤ −0.1). The strongest negative correlation was observed between self-concept and anxiety (*r* = −0.75).

**TABLE 1:** Descriptive statistics.

Variable	*N*	Mean	*SD*	Skewness	Kurtosis	Median	Min	Max
MAC	1,464	489.94	85.34	0.22	–0.32	484.97	271.23	765.47
Age	1,464	15.82	0.28	–0.03	–1.14	15.83	15.33	16.33
MB	1,464	1.54	0.53	1.80	3.79	1.38	1.00	4.00
INMM	1,464	2.97	0.74	–0.59	0.19	3.00	1.00	4.00
MA	1,464	2.35	0.71	0.19	–0.24	2.40	1.00	4.00
MI	1,464	2.37	0.80	0.14	–0.60	2.25	1.00	4.00
MPC	1,464	3.08	0.48	0.04	–0.42	3.00	1.50	4.00
MSC	1,464	2.71	0.76	–0.14	–0.61	2.80	1.00	4.00
MP	1,464	3.57	0.76	–0.17	–0.15	3.60	1.00	5.00
SES	1,464	0.19	0.97	–0.29	–0.25	0.27	–3.80	2.60

*MAC, math achievement; MB, math behavior; INMM, instrumental motivation in math; MA, math anxiety; MI, interest in math; MPC, math perceived control; MSC, math self-concept; MP, persistence.*

**TABLE 2:** Correlation between variables.

	MI	MPC	MSC	MA	INMM	MB	MP	MAC
MI	1							
MPC	0.36	1						
MSC	0.65	0.55	1					
MA	–0.46	–0.57	–0.75	1				
INMM	0.63	0.45	0.47	–0.34	1			
MB	0.41	0.06	0.31	–0.1	0.29	1		
MP	0.38	0.38	0.44	–0.4	0.33	0.21	1	
MAC	0.15	0.36	0.43	–0.45	0.16	0.01	0.26	1

*MAC, math achievement; MB, math behavior; INMM, instrumental motivation in math; MA, math anxiety; MI, interest in math; MPC, math perceived control; MSC, math self-concept; MP, persistence.*

Confirmatory factor analysis was used to interrogate the validity of measurement constructs for anxiety, instrumental motivation, interest, self-concept, and perceived control (Supplementary Figure 1). Fitted model has significant Chi-square test (χ^2^ = 2,489.822, *p* < 0.001). The Comparative Fit Index (CFI) is 0.907. The Standardized Root Mean Residual (SRMR) is 0.071. The Root Mean Square Error of Approximation (RMSEA) is 0.077. All the fitting statistics show that the CFA model was just acceptable (Wang and Wang, 2009). This is mainly due to the construct of perceived control, which shows a high residual correlation with other items, especially from anxiety and self-concept (Supplementary Figure 2). Overall, CFA indicates that the data fits a proposed measurement model for these five variables.

Latent profile analysis was used to explore latent subgroups of individuals defined by five constructs: anxiety, instrumental motivation, interest, self-concept, and perceived control. Considering different variance and evident correlations among the five variables, models with 14 different constraints on variance–covariance matrix structure were included for comparison, from one latent subgroup to 12 latent subgroups. Different structures for the variance–covariance matrix are represented by three letters, indicating “scale,” “shape,” and “orientation” of latent multivariable distribution, respectively (Scrucca et al., 2016). Bayesian information criterion (BIC) and Integrated Complete-data Likelihood (ICL, which is BIC penalized by estimated mean entropy) are used first to compare all 168 models (Supplementary Figures 3A,B) (Biernacki et al., 2000). Both criteria showed that the “VVE” model (varying variances and equal correlations) with 10 latent subgroups are the best. The likelihood ratio test (LRT) for assessing the number of latent subgroups was performed. Again, the VVE model with 10 subgroups was significant compared with the VVE model with 9 latent subgroups. However, the VVE model with 11 subgroups was not significant compared to the VVE model with 10 subgroups. The model with 10 subgroups had a normalized entropy (*E*_norm_, equation 1) of 0.822. The VVE models with 9 and 12 subgroups have the second and third highest BIC. The normalized entropies were both 0.79, lower than that of the model with 10 subgroups. Above all, the VVE model with 10 subgroups demonstrated the best model fit and was chosen for further analysis. The smallest subgroup includes 2.5% of all individuals (seen in Table 3).

**TABLE 3:** Latent profile analysis (LPA) fitting results.

Parameters	Values
Bayesian Information Criterion (BIC)	−16,458.430
Integrated Complete-data Likelihood (ICL)	−17,040.510
Bootstrap Likely Ratio Test (LRT) vs. 9	0.001
Bootstrap Likely Ratio Test (LRT) vs. 11	0.187
Normalized entropy	0.822
minGroupPct	0.020

**FIGURE 1:**
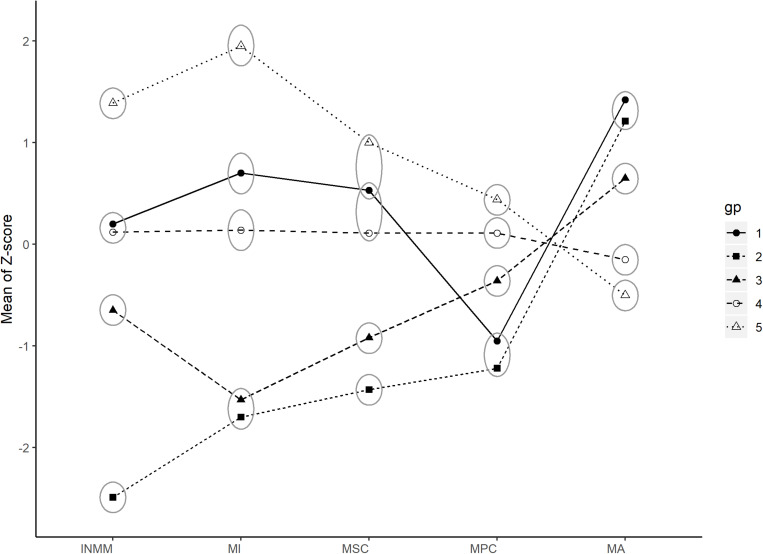
Latent subgroups of students from latent profile analysis (LPA) and secondary clustering. Means of residualized *Z* scores of INMM, MI, MSC, MPC, and MA are shown for each latent subgroup. Ellipse indicates a non-significant difference between these subgroups of certain variables. Group 1—high math anxiety and medium-high motivation. Group 2—high math anxiety and low motivation. Group 3—medium math anxiety and medium-low motivation. Group 4—low math anxiety and medium motivation. Group 5—low anxiety and high motivation. INMM, instrumental motivation in math; MA, math anxiety; MI, interest in math; MPC, math perceived control; MSC, math self-concept; Gp, latent subgroups. Significant level at 5%.

The 10-group model provided the best BIC and ICL. There were some subgroups with very similar patterns (Supplementary Figure 4). To make our results more interpretable, we conducted secondary clustering (Baudry et al., 2010). This secondary clustering procedure has no assumptions on underlying distributions; it solely focuses on reducing entropies by combining severely overlapped groups. Entropies (*E*, equation 2) of results with 1 to 10 subgroups were plotted, and piecewise linear regression was used to find the “elbow” point where decreasing of entropy becomes less (Supplementary Figure 5). Two elbow points at seven groups and five groups were found, and the clustering with five resultant subgroups was chosen for interpretability and simplicity (seen in Figure 1 and Table 4).

**TABLE 4:** Latent subgroup means.

Group	*N*	INMM	MI	MSC	MPC	MA
1	33	0.20	0.70	0.53	–0.95	1.42
2	63	–2.49	–1.70	–1.43	–1.22	1.21
3	139	–0.65	–1.53	–0.92	–0.36	0.65
4	1,159	0.12	0.14	0.11	0.11	–0.15
5	70	1.39	1.95	1.00	0.44	–0.50

*INMM, instrumental motivation in math; MA, math anxiety; MI, interest in math; MPC, math perceived control; MSC, math self-concept. Group 1-high math anxiety and medium-high motivation. Group 2-high math anxiety and low motivation. Group 3-medium math anxiety and medium-low motivation. Group 4-low math anxiety and Medium motivation. Group 5-low anxiety and high motivation.*

Class 1—high math anxiety and medium-high motivation. The size of this subgroup was the smallest among all five subgroups with approximately 2.3% of all students (*n* = 33). About 57.6% of the students in this subgroup were males (*n* = 19) and 42.4% were females (*n* = 14). The SES mean was the lowest of all the five subgroups. The students in this subgroup were characterized by the highest levels of math anxiety among all the subgroups and medium-high motivation, except for perceived control that is low.

Class 2—high math anxiety and low motivation. The size of this subgroup was the second smallest among all the subgroups with around 4.3% of the students (*n* = 63). About 54% of the students in this subgroup were males (*n* = 34) and 46% were females (*n* = 29). The SES mean of this subgroup was the highest of all subgroups. The students in this subgroup were characterized by very high math anxiety, the lowest MFs. The math anxiety levels of the students in this subgroup showed no significant difference from the math anxiety levels of the students in class 1, but the MFs levels of the students in this subgroup were significantly lower than the MFs levels in subgroup 1.

Class 3—medium math anxiety and medium-low motivation. The size of this subgroup was medium with approximately 9.4% of the students (*n* = 139). About 47.5% of the students in this subgroup were males (*n* = 66) and 52.5% were females (*n* = 73). The mean SES of this subgroup was very close to the mean SES of all 1,464 students (0.192). The students in this subgroup were characterized by medium math anxiety, a medium level of motivation and perceived control, and a low level of interest and self-concept.

Class 4—low math anxiety and medium motivation. The size of this subgroup is the largest with approximately 79.2% of the students (*n* = 1,159). About 50.5% of the students in this subgroup were males (*n* = 585) and 49.5% were females (*n* = 574). Mean SES is very close to the mean SES of all 1,464 students. The students in this subgroup were characterized by low math anxiety and medium MFs.

Class 5—low math anxiety and high motivation. The size of this subgroup is relatively small with approximately 4.8% of the students (*n* = 70). About 55.7% of the students in this subgroup were males (*n* = 39) and 44.3% were females (*n* = 31). The SES mean of this subgroup was higher than that of classes 1, 3, and 4, but lower than that of class 2. The students in this subgroup were characterized by the lowest anxiety among all the subgroups and the highest MFs among all the subgroups.

Math achievement of students from all five subgroups was compared using linear regression with gender and SES as covariates (adjusted *R*^2^ = 0.201). In this model, male students performed significantly better than female students (χ^2^ = 18.06, *p* < 0.001). SES significantly positively associates with math achievement (χ^2^ = 261.15, *p* < 0.001). Math achievement is significantly associated with subgroup membership with a small effect size (*f*^2^ = 0.039, χ^2^ = 66.90, *p* < 0.001) (Cohen, 1992). We used a pairwise comparison to determine the differences in math achievement between subgroups. As the results shown in Table 5, among all the five subgroups, the classes 4 and 5 have the highest achievements, significantly higher than classes 1 and 2. Both classes 4 and 5 have higher achievements than that of class 3, but only class 4 reached significance. There are no significant differences in math achievement between classes 4 and 5. Class 1 has the lowest math achievement.

**TABLE 5:** Subgroup comparison.

Group	*N*	Male	Female	Male (%)	SES	log(MB)	MP	MAC
1	33	19	14	57.6	–0.037	0.72 (c)	3.25 (a)	407.5 (a)
2	63	34	29	54.0	0.252	0.19 (a)	3.05 (a)	448.4 (ab)
3	139	66	73	47.5	0.192	0.23 (a)	3.21 (a)	473.6 (bc)
4	1,159	585	574	50.5	0.193	0.38 (b)	3.62 (b)	496.5 (d)
5	70	39	31	55.7	0.234	0.68 (c)	4.09 (c)	490.4 (cd)

*MAC, math achievement; MB, math behavior; MP, persistence. Group 1-high math anxiety and medium-high motivation. Group 2-high math anxiety and low motivation. Group 3-medium math anxiety and medium-low motivation. Group 4-low math anxiety and medium motivation. Group 5-low anxiety and high motivation. Letters in parenthesis of the last three columns are indicating significance of comparing means between different groups correcting for multiple testing. If two groups share any same letter, the means of MB, MP, or MAC are not significantly different. If there is no shared letter between two groups, then the means of MB, MP, or MAC are significantly different.*

Math-related behaviors of students among all the five subgroups were compared using linear regression with gender and SES as covariates (adjusted *R*^2^ = 0.147). Male students showed that they were significantly more frequently involved in math-related activities than female students (χ^2^ = 33.14, *p* < 0.001) and SES positively associates with strong persistence (χ^2^ = 15.70, *p* < 0.001). Math-related behaviors also were significantly associated with subgroup membership with a small effect size (*f*^2^ = 0.137, χ^2^ = 193.55, *p* < 0.001). Results of pairwise comparison for behavior among subgroups are shown (seen in Table 5). Classes 1 and 5 showed the highest frequency of math behavior and there was no significant difference of math behavior between them. No significant difference was found between math behavior of classes 4 and 3, which showed less frequency than math behavior of classes 1 and 5. Class 2 demonstrated the least frequent math-related behavior.

Students’ persistence in learning math among all five subgroups were compared using linear regression with gender and SES as covariates (adjusted *R*^2^ = 0.093). Male students showed significantly stronger persistence than female students (χ^2^ = 9.64, *p* = 0.002), and SES positively associates with strong persistence (χ^2^ = 31.10, *p* < 0.001). Persistence is also significantly associated with subgroup membership with a small effect size (*f*^2^ = 0.076, χ^2^ = 110.79, *p* < 0.001). Through pairwise comparison, we found that class 5 showed the highest level of persistence, significantly higher than all other subgroups. Class 4 has the second-highest level of persistence, significantly higher than classes 1, 2, and 3. There was no significant difference among the persistence of classes 1, 2, and 3 (Table 5).

To sum up, we found five subgroups of students with different patterns of motivational and affective factor (math anxiety). Significant differences in math behavior, persistence, and achievement were found among subgroups.

## Discussion

The main goal of our study was to investigate the latent profiles of the students with respect to five non-cognitive factors: math interest, math self-concept, perceived control in math, instrumental motivation to learn math, and math anxiety. We also sought to investigate the relationship between group classification and mathematics achievement, persistence, and behavior.

### Latent Profile Analysis and Secondary Clustering

To identify the profiles of students, we employed LPA. For getting practically interpretable results, we adopted the secondary clustering technique to reduce group numbers. This method helps us merge subgroups overlapping significantly in order to avoid ambiguity of classification of students. In practice, the resultant five subgroups are easier to be applied compared with the initial 10 classes suggested by BIC criteria to a small group of students such as a class or a school. Most of the time, BIC criteria would generate appropriate results in the latent profile analysis. However, due to the assumption about underlying distribution (multivariate gaussian in our case), BIC could report overlapping clusters without warning. ICL took into account entropy to punish overlapping clusters (Biernacki et al., 2000). ICL could be viewed as BIC with an extra term of entropy. This means the weight of entropy in ICL could not be adjusted, and it also could potentially return too many clusters when the underlying distribution does not match our assumptions. In other words, the BIC criterion is seeking for underlying distributions other than clusters. ICL punishes less on deviation from distribution assumption but still depends on it. In our study, both BIC and ICL provided us with 10 subgroups as the best results. However, some subgroups showed very similar patterns in means (Supplementary Figure 4). To collapse these closely related subgroups, we further clustered these 10 subgroups into five subgroups, purely based on entropy (Baudry et al., 2010).

Overall, we found five distinct latent profiles that describe US students’ motivation and affection. According to our results, except for class 1, the other four subgroups all show a positive correlation among motivational variables. Indeed, Trautwein et al. (2012) found that students who believed that they had high ability in an academic domain perceived high task values in that domain. In addition, except for class 1, the other four subgroups all showed a seemingly negative correlation between motivational variables and anxiety. This is consistent with previous variable-centered studies that suggested an inverse relationship between math anxiety and motivational variables (Meece et al., 1990; Ahmed et al., 2012). A recent study employing ACE model on 3,410 twins showed negative correlations between math anxiety and two math attitude variables, interest and self-efficacy, both phenotypically (−0.45) and genetically (−0.7) (Malanchini et al., 2020). Interestingly, with a person-centered approach, our study found an exceptional subgroup, class 1, of which the students with medium math interest, instrumental motivational to learn, and math self-concept had high math anxiety and low rather than medium perceived control in math. Compared with other subgroups, these students were probably extremely anxious about learning mathematics. They believed in their mathematics ability to some extent and perceived that mathematics was enjoyable to learn and valuable for their future studies and careers, but they doubted whether they can control their ability to succeed in math.

Our findings provide distinguishing profiles of students based on motivational and affective factors. This will help teachers to understand the diversity of the students’ math motivation and affection. Moreover, identifying students’ subgroups and the understanding of their characteristics are beneficial for teachers who attempt to customize an educational plan with ideal instruction type and teaching strategies for specific groups of students. Oberlin (Bonijoly et al., 1982) posited that an identical teaching method for the entire class would cause anxiety. Thus, it is important for teachers to identify the subgroups of students by their psychological characteristics to facilitate differential teaching based on students’ needs. It is helpful if teachers identify groups of students who are potentially at risk of failure and give those students appropriate instruction.

To better understand the characteristics of the students’ in different subgroups, we further investigated the differences in mathematics achievement, persistence, and math behaviors (math-related activities) using regression. In the regression models, we used gender and SES as covariates, for a number of studies have revealed that gender and SES affect students’ math achievement, persistence, and math behaviors (Eccles and Wigfield, 2002; Passolunghi et al., 2014; Barron and Hulleman, 2015).

### Differences in Math Behaviors Across Profiles

Using regression, we found that there are differences among subgroups concerning math-related behaviors. Math behavior was measured by eight items with values bounded between one and four. Moreover, math behavior values are strongly right-skewed, which was partially corrected by logarithm transformation. Diagnostic plots of the regression model showed no digress from the normality assumption (data not shown). The regression effect size (*f*^2^ = 0.137) of subgroups on math behavior is small (0.02–0.15) according to Cohen (1992). Interestingly, subgroup covariate boosted *R*^2^ from 0.03 to 0.15, indicating that our latent subgroups explained much more variation in math behavior than gender and SES in the linear regression model. This is different from math achievement, where gender and SES explain a significant portion of variation (see below). As the pairwise comparison results show, the students in classes 1 and 5 reported the most frequently involved in math-related activities, followed by classes 4, 3, and 2 (no significant differences were found between classes 3 and 2). Without regard to class 1, the other subgroups of students with higher motivation and lower math anxiety more frequently participated in math-related activities, like “talk about mathematics problems with my friends” and “take part in mathematics competitions.” This finding agrees with previous studies that demonstrated that academic motivation directly or indirectly positively affects math-related activities (Rotgans and Schmidt, 2009; Green et al., 2012). However, given the existence of class 1 of which students were highly anxious with medium motivation but low perceived control, we assume that the controllability of math and negative emotion probably did not affect the involvement of certain students in math-related activities. This reveals that math self-concept, math interest, and instrumental motivation to learn math are more significant than math anxiety and perceived control in math to determine math behaviors.

### Differences in Persistence Across Profiles

We also found significant differences in persistence across five subgroups of United States students with a small effect size (*f*^2^ = 0.076). Similar to math behavior, persistence is bounded by one and five. However, persistence values are not skewed. In our multiple regression model, SES and gender alone explain less than 3% variation of math persistence (*R*^2^ = 0.026); adding subgroups as covariate boosted *R*^2^ to 0.097, indicating that math persistence is barely determined by gender or SES, but more determined by subgroups. The students in class 5, which is characterized by the lowest math anxiety and highest motivation, tended to be the most persistent, followed by classes 4, 3, 2, and 1, there was no significant difference found among classes 1–3. As the results show, students who are highly motivated and less anxious are more likely to persist when facing difficulties and challenges without considering the unique subgroup, class 1. The findings are consistent with the prior findings that higher levels of perceived competence and subjective task values predicted higher levels of persistence (Jacobs et al., 1984; Lavigne et al., 2007). However, regarding the students in class 1, they tended to avoid challenging themselves when facing difficulties regardless of relatively high instrumental motivation, interest, and self-concept. It is possible that the negative emotion diminishes students’ willingness to engage, even if they perceive these tasks useful and enjoyable, and themselves competent (Eccles and Wigfield, 2002; Jiang et al., 2018).

### Differences in Mathematics Achievement Across Profiles

The results of the study indicate that profile membership was significantly related to the United States students’ mathematics achievement with a small effect size (*f*^2^ = 0.039). Contrasting to what we observed on math behavior and persistence, gender and SES explains around 20% of the variation in math achievement (*R*^2^ = 0.204). We found that the mathematics achievement of classes 4 and 5 was the highest of all, followed by classes 3, 2, and 1. Based on our findings, the subgroup with higher motivation and lower math anxiety had higher mathematics achievement than the subgroup with lower motivation and higher math anxiety except for class 1. This is supported by the previous study, which also employed latent profile analysis and found that a combination of lower motivation and higher math anxiety suggested lower math achievement (Wang et al., 2018). Our findings are also supported by previous studies, which investigate motivational and affective variables separately. The studies show perceived competence positively affected academic achievement (You et al., 2011; Petersen and Hyde, 2017). Besides, Murayama et al. (2013) demonstrated that perceived control, extrinsic motivation, and intrinsic motivation positively predicted subsequent mathematics achievement.

The unexpected finding of class 1 shows that a subgroup of students with a medium level of math interest, instrumental motivation, and math self-concept, but low perceived control and high math anxiety had low mathematics achievement. This indicates that the students with comparatively high motivation may not reach relatively high math achievement, if they have extremely high math anxiety, suggesting that math anxiety negatively affects mathematics achievement. Indeed, while the students are motivationally engaging in learning, high MA may interfere cognitive engagement by disturbing working memory (Ashcraft, 2002); therefore, they may perform poorly in math assessment (Ramirez et al., 2016).

Overall, the significant differences in mathematics achievement show that five latent profiles not only describe patterns of students’ motivation and affection, but also can be interpreted as performance-based subgroups of students. We found that there were lower achievers with high math anxiety regardless of the motivation level they had. Teachers should pay more attention to these students, for they may demonstrate that they are comparatively confident and both intrinsically and extrinsically motivated to learn math, but still perform poorly. The possible reason lies in that, although these students may invest time and efforts in learning due to their comparatively high motivation, the math anxiety may cause dysfunction of working memory, which impairs learning outcomes (Ramirez et al., 2018). However, it is probably difficult for teachers to identify these students merely based on observation of their behaviors in learning without referring to their test scores.

We observed significant differences among different subgroups of math behavior, math persistence, and mathematics achievement. They share certain patterns such as students with high motivation and low anxiety are more involved in math activities, more persistent when facing math challenges, and achieved higher. However, there are some differences: there are students whose math behaviors are more driven by math self-concept, instrumental motivation, and interest, while are less influenced by math perceived control and anxiety. For other groups of students, math persistence and mathematics achievement are more affected or even dominated by math anxiety and/or math perceived control. This could be due to the fact that math behavior measures more the involvement of activities, which normally does not incur pressure on participants, which will not drive away students who fear doing other challenging math tasks due to high math anxiety or low perceived control, like the students we observed in class 1.

In conclusion, our findings reveal the complexity of the students’ motivational and affective profiles. We found five subgroups of the United States students based on motivational and affective factors. Consistent with a number of previous studies, four out of five subgroups of students demonstrated a negative relationship between motivation and math anxiety. Interestingly, one subgroup showed both medium to high motivation and very high anxiety at the same time. In addition, our findings show that students with frequent involvement in math activities had high self-concept, instrumental motivation, and math interest, while students with a high level of persistence and math achievement are characterized by low math anxiety and high perceived control in math. Our findings are significant for teachers and educators to understand the diversity of students and provide theoretical and practical support for individualized and differentiated instruction.

## Limitation

There are several limitations to our study. First, the self-reported math behavior scale does not cover all math-related behaviors in reality. Using interviews or observations to capture more information about students’ math-related behaviors are suggested in future research. Second, we only used data from 1,464 students who have no missingness among all used items in this study. The sample size of 1,464 is still larger than other studies of similar design (Hart et al., 2016; Wang et al., 2017; Wang et al., 2018). In addition, due to the fact that students are receiving different subsets of questions of PISA questionnaire by rotated test design (OECD, 2013) means we can assume MCAR. Under this assumption, our approach (LD) will not bring bias to the following analysis (see Supplementary Section “Missingness”). To further validate our results using LD, we imputed missing data from 1,640 students five times. A similar analysis was conducted on these five imputed datasets and similar results were found (for details, please see Supplementary Section “Partial Multiple Imputation Using 3,104 Students Showed Similar Results”). However, the sample size could be potentially increased by using appropriate imputation methods (such as FIML) under some reasonable assumptions about the pattern of missingness. Third, the process of determining the final number of groups of secondary clustering was partially subjective. Even though we used piecewise linear regression to assist in finding elbow points, deciding the number of clusters in this process is still subjective. To our knowledge, there is no formal statistical testing that can be used to determine optimal clusters. Last, our study only focused on the United States sample. Thus, the results should be extended to students from other countries with caution. The future study could attempt to use cross-national data to increase power (detecting more rare subgroups), compare students’ subgroups across countries.

## Data Availability Statement

The datasets generated for this study are available on request to the corresponding author.

## Ethics Statement

The data included in this study was derived from PISA. Therefore, the study did not require ethics approval.

## Author Contributions

FX made substantial contributions to the conception and design of the study, data analysis, and interpretation. LS made substantial contributions to the data analysis and interpretation. Both authors contributed to the article and approved the submitted version.

## Conflict of Interest

The authors declare that the research was conducted in the absence of any commercial or financial relationships that could be construed as a potential conflict of interest.
