# Supplementary material for: Students’ Motivation and Affection Profiles and Their Relation to Mathematics Achievement, Persistence, and Behaviors
Source: Front Psychol. 2021 Jan 14;11:533593. doi: 10.3389/fpsyg.2020.533593 (PMC7841336; doi:10.3389/fpsyg.2020.533593)
Supplement: Supplementary file 1 [file Data_Sheet_1.docx]

Supplementary Material

# Supplementary Data

## Missingness

Among 4978 students, considering 37 response items to 7 variables used in our study (anxiety, self-concept, instrumental motivation, interest, perceived control, behavior, and persistence), there are 184186 items in total. 119197 (64.7%) of them are not missing. 64989 (35.3%) items are missing. Among all missing items, 62292 (95.8%) are N/A (represented by 7 in PISA), which is caused by non-student’s reason, in our case, the ‘questionnaire rotation’. According to PISA 2012 Technical Report, students miss anxiety and self-concept completely received form A, students miss instrumental motivation, interest, perceived control, behavior, and persistence items received form C, and other students received form B. (see page 61 of PISA 2012 technical report, <https://www.oecd.org/pisa/pisaproducts/PISA-2012-technical-report-final.pdf>). 29 are invalid values (represented by 8 in PISA). 2668 are item-wise missing caused by participants (represented by 9 in PIS). Visualize missing patterns of 3 missing types in following figure:


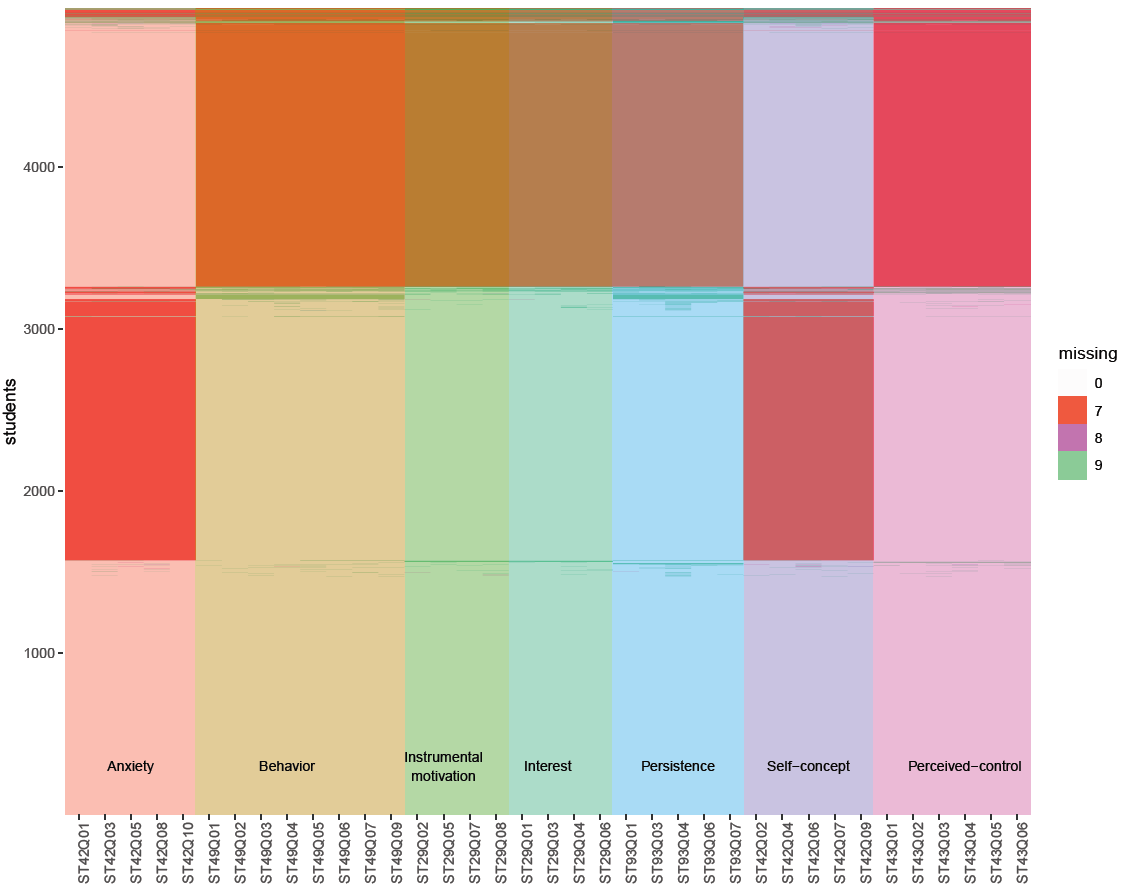


Item-wise missing percentages are listed in following table:

| **construct** | **item** | **missing_7(%)** | **missing_8(%)** | **missing_9(%)** |
| --- | --- | --- | --- | --- |
| anxiety | ST42Q01 | 33.7 % | 0 % | 1.1 % |
|  | ST42Q03 | 33.7 % | 0 % | 1.5 % |
|  | ST42Q05 | 33.7 % | 0.1 % | 1.3 % |
|  | ST42Q08 | 33.7 % | 0 % | 1.4 % |
|  | ST42Q10 | 33.7 % | 0 % | 1.3 % |
| behaviour | ST49Q01 | 33.9 % | 0 % | 1.6 % |
|  | ST49Q02 | 33.9 % | 0 % | 1.7 % |
|  | ST49Q03 | 33.9 % | 0 % | 1.8 % |
|  | ST49Q04 | 33.9 % | 0 % | 2 % |
|  | ST49Q05 | 33.9 % | 0 % | 1.9 % |
|  | ST49Q06 | 33.9 % | 0 % | 1.8 % |
|  | ST49Q07 | 33.9 % | 0 % | 1.9 % |
|  | ST49Q09 | 33.9 % | 0 % | 2 % |
| Instrumental Motivation | ST29Q02 | 33.9 % | 0 % | 0.8 % |
|  | ST29Q05 | 33.9 % | 0 % | 0.9 % |
|  | ST29Q07 | 33.9 % | 0 % | 0.9 % |
|  | ST29Q08 | 33.9 % | 0.1 % | 1 % |
| interest | ST29Q01 | 33.9 % | 0 % | 0.7 % |
|  | ST29Q03 | 33.9 % | 0 % | 0.8 % |
|  | ST29Q04 | 33.9 % | 0 % | 1 % |
|  | ST29Q06 | 33.9 % | 0 % | 1 % |
| Persistence | ST93Q01 | 33.9 % | 0 % | 1.9 % |
|  | ST93Q03 | 33.9 % | 0 % | 2 % |
|  | ST93Q04 | 33.9 % | 0 % | 2.6 % |
|  | ST93Q06 | 33.9 % | 0 % | 2 % |
|  | ST93Q07 | 33.9 % | 0 % | 1.9 % |
| self-concept | ST42Q02 | 33.7 % | 0 % | 1.2 % |
|  | ST42Q04 | 33.7 % | 0.1 % | 1.2 % |
|  | ST42Q06 | 33.7 % | 0.1 % | 1.4 % |
|  | ST42Q07 | 33.7 % | 0 % | 1.3 % |
|  | ST42Q09 | 33.7 % | 0 % | 1.3 % |
| self-control | ST43Q01 | 33.9 % | 0 % | 1.3 % |
|  | ST43Q02 | 33.9 % | 0 % | 1.2 % |
|  | ST43Q03 | 33.9 % | 0 % | 1.5 % |
|  | ST43Q04 | 33.9 % | 0 % | 1.5 % |
|  | ST43Q05 | 33.9 % | 0 % | 1.4 % |
|  | ST43Q06 | 33.9 % | 0 % | 1.4 % |

## Partial Multiple Imputation using 3104 students showed similar results

To further show that LD would not bias our study, we have tried following (“partial MI” with no pooling step):

1. Instead of maintaining all cases, we get rid of cases missing 5 out of 7 constructs used in this study completely (The top 1/3 of the figure above). These students simply contain too less information (only anxiety and self-concept). Then we have 3104 students left.
2. Imputation of missing data using EM algorithm (Schafer, 1997) with ‘imputeData’ function from ‘mclust’ package of R.
3. Repeat step 2 for 5 times to generate 5 different imputed datasets with 3104 students each.
4. Run LPA on each of these 5 datasets, choosing model with BIC, ICL, and entropy criteria as we did for LD data in manuscript. Similar to what we found before, for all 5 imputed datasets, VVE model with 10 to 12 subgroups fit the data the best.
5. Secondary clustering was used too. But we didn’t select number of subgroups using ‘elbow method’ like before. Instead, we fixed it to 5 so it is easier to compare with results in manuscript. Interestingly, when we have done this, we rediscovered 4 of 5 groups which were identified when using data with no missing. There is a new group not revealed before. After we increased the subgroup number to 6 with secondary clustering. In all 5 imputed datasets, we could find all five subgroups we have discovered using LD (except for the group 3 in imputation 2, which shows slightly different pattern comparing to corresponding group in original results). Please see figures and tables below:


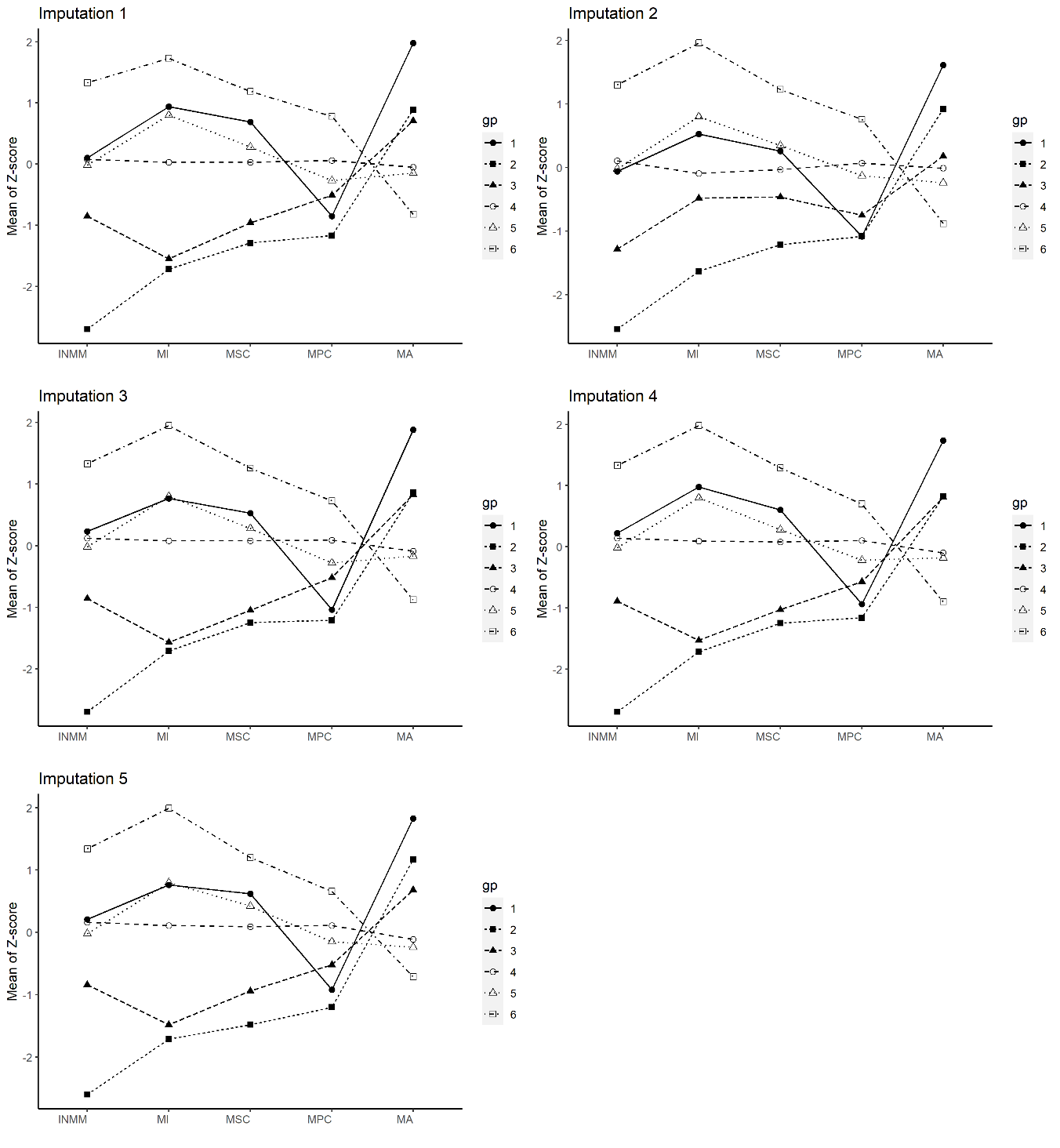


Means of all 7 variables of all groups in 5 imputation data sets are shown below. gp: group, n: number of students, imr: instrumental motivation, ir: interest, scr: self-concept, scor: perceived control, ar: anxiety, b: behavior in math, p: math persistence, ach: achievement in math.

Table: LPA subgroup results from imputation 1

| **gp** | **n** | **imr** | **ir** | **scr** | **scor** | **ar** | **b** | **p** | **ach** |
| --- | --- | --- | --- | --- | --- | --- | --- | --- | --- |
| 1 | 23 | 0.10 | 0.94 | 0.69 | -0.85 | 1.98 | 2.33 | -0.53 | 396 |
| 2 | 90 | -2.70 | -1.72 | -1.29 | -1.17 | 0.89 | -0.52 | -0.80 | 452 |
| 3 | 325 | -0.85 | -1.55 | -0.96 | -0.51 | 0.71 | -0.50 | -0.47 | 465 |
| 4 | 2267 | 0.08 | 0.03 | 0.03 | 0.06 | -0.05 | -0.07 | 0.01 | 490 |
| 5 | 141 | -0.02 | 0.80 | 0.28 | -0.27 | -0.15 | 0.44 | 0.07 | 457 |
| 6 | 258 | 1.33 | 1.73 | 1.19 | 0.78 | -0.82 | 0.95 | 0.78 | 516 |

- Table: LPA subgroup results from imputation 2

| **gp** | **n** | **imr** | **ir** | **scr** | **scor** | **ar** | **b** | **p** | **ach** |
| --- | --- | --- | --- | --- | --- | --- | --- | --- | --- |
| 1 | 44 | -0.06 | 0.53 | 0.26 | -1.08 | 1.61 | 1.12 | -0.41 | 414 |
| 2 | 145 | -2.54 | -1.63 | -1.21 | -1.08 | 0.92 | -0.51 | -0.66 | 455 |
| 3 | 98 | -1.28 | -0.48 | -0.46 | -0.75 | 0.18 | -0.36 | -0.50 | 472 |
| 4 | 2475 | 0.11 | -0.09 | -0.03 | 0.07 | -0.01 | -0.08 | 0.00 | 489 |
| 5 | 163 | -0.02 | 0.80 | 0.35 | -0.13 | -0.24 | 0.43 | 0.06 | 463 |
| 6 | 179 | 1.30 | 1.96 | 1.23 | 0.76 | -0.88 | 1.11 | 0.81 | 517 |

- Table: LPA subgroup results from imputation 3

| **Gp** | **n** | **imr** | **ir** | **scr** | **scor** | **ar** | **b** | **p** | **ach** |
| --- | --- | --- | --- | --- | --- | --- | --- | --- | --- |
| 1 | 24 | 0.23 | 0.77 | 0.53 | -1.04 | 1.88 | 1.87 | -0.57 | 402 |
| 2 | 87 | -2.70 | -1.71 | -1.25 | -1.21 | 0.86 | -0.52 | -0.82 | 456 |
| 3 | 321 | -0.86 | -1.57 | -1.05 | -0.52 | 0.83 | -0.52 | -0.44 | 464 |
| 4 | 2358 | 0.12 | 0.08 | 0.08 | 0.09 | -0.09 | -0.04 | 0.03 | 491 |
| 5 | 146 | -0.02 | 0.80 | 0.28 | -0.28 | -0.17 | 0.45 | 0.09 | 456 |
| 6 | 168 | 1.33 | 1.95 | 1.26 | 0.73 | -0.88 | 1.11 | 0.85 | 515 |

- Table: LPA subgroup results from imputation 4

| **Gp** | **n** | **imr** | **ir** | **scr** | **scor** | **ar** | **b** | **p** | **ach** |
| --- | --- | --- | --- | --- | --- | --- | --- | --- | --- |
| 1 | 28 | 0.22 | 0.98 | 0.60 | -0.94 | 1.74 | 1.97 | -0.42 | 404 |
| 2 | 88 | -2.70 | -1.72 | -1.25 | -1.16 | 0.82 | -0.50 | -0.75 | 453 |
| 3 | 335 | -0.89 | -1.53 | -1.03 | -0.57 | 0.81 | -0.51 | -0.48 | 463 |
| 4 | 2345 | 0.14 | 0.09 | 0.08 | 0.10 | -0.10 | -0.03 | 0.04 | 491 |
| 5 | 154 | -0.02 | 0.80 | 0.28 | -0.22 | -0.18 | 0.45 | 0.07 | 460 |
| 6 | 154 | 1.33 | 1.98 | 1.29 | 0.70 | -0.90 | 1.10 | 0.87 | 513 |

- Table: LPA subgroup results from imputation 5

| **gp** | **n** | **imr** | **ir** | **scr** | **scor** | **ar** | **b** | **p** | **ach** |
| --- | --- | --- | --- | --- | --- | --- | --- | --- | --- |
| 1 | 26 | 0.21 | 0.76 | 0.62 | -0.92 | 1.83 | 1.94 | -0.44 | 401 |
| 2 | 105 | -2.60 | -1.71 | -1.48 | -1.20 | 1.17 | -0.54 | -0.82 | 450 |
| 3 | 343 | -0.84 | -1.48 | -0.94 | -0.52 | 0.68 | -0.48 | -0.44 | 465 |
| 4 | 2321 | 0.16 | 0.11 | 0.09 | 0.11 | -0.11 | -0.03 | 0.04 | 492 |
| 5 | 161 | -0.02 | 0.80 | 0.42 | -0.15 | -0.24 | 0.43 | 0.11 | 463 |
| 6 | 148 | 1.34 | 1.99 | 1.20 | 0.66 | -0.71 | 1.20 | 0.85 | 510 |

Generally, we re-discovered 5 subgroups which were identified using LD. There was a new group identified (group 3 in above plots) which is not surprising considering we were using 200% data. Most importantly, the most interesting group, group 1, was identified in all imputations. By looking at the 3 outcome variables (behavior, persistence, and achievement) used in our manuscript, we could achieve similar conclusions too, such as that students with high motivation and low anxiety were more involved in math activities, more persistent when facing math challenges, with exceptions of students from group 1 who had very high math behavior scores.

We call this ‘partial MI’ because there is no pooling step. We manually inspected results from all 5 imputations. This is because there is no well-defined pooling method of MI for LPA analysis yet.

# Supplementary Figures and Tables

## Supplementary Figures


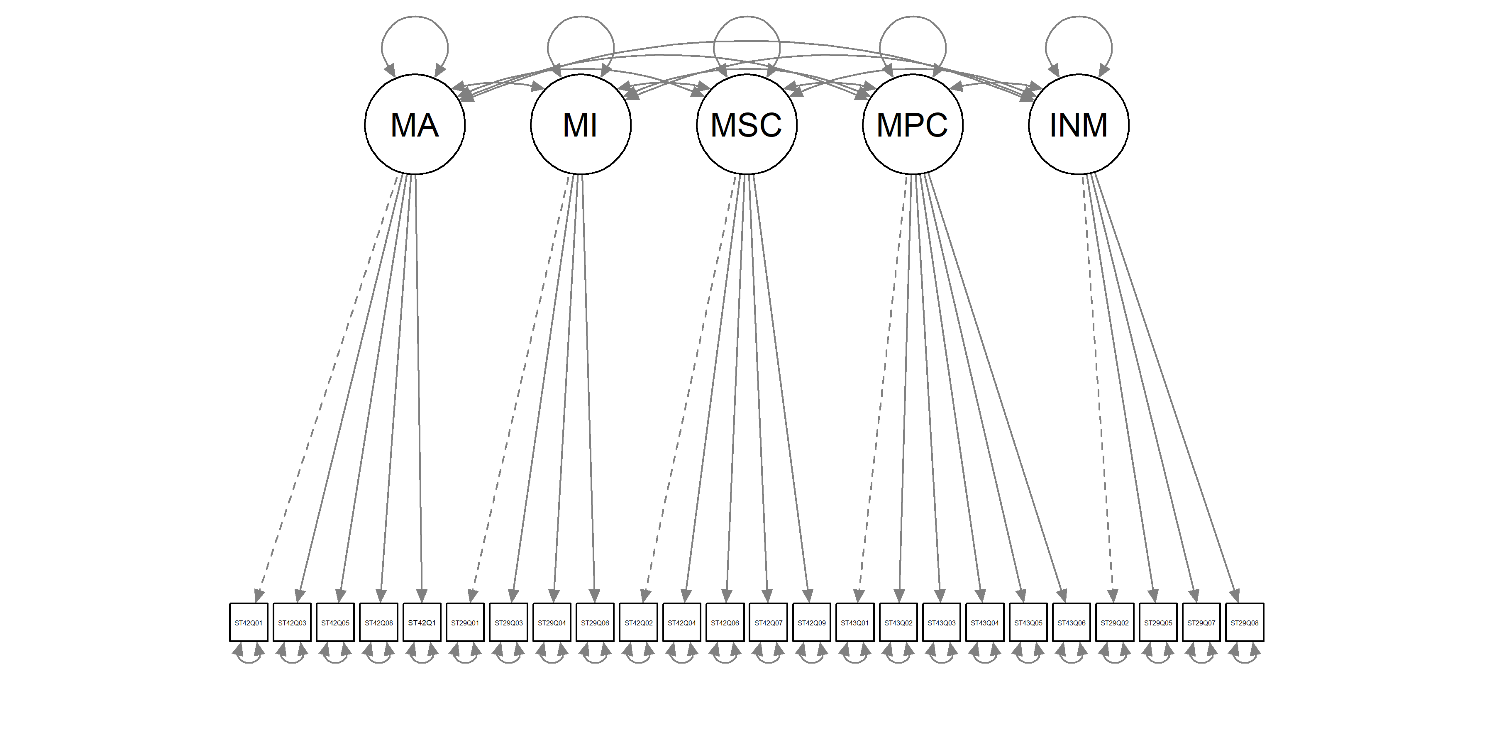


**Supplementary Figure 1.** Path diagram of CFA model with all possible covariances enabled. MA, math anxiety. MI, interest in math. MSC, math self-concept. MPC, math perceived control. INM, instrumental motivation of math. ST**Q**, response item names from PISA 2012.

**
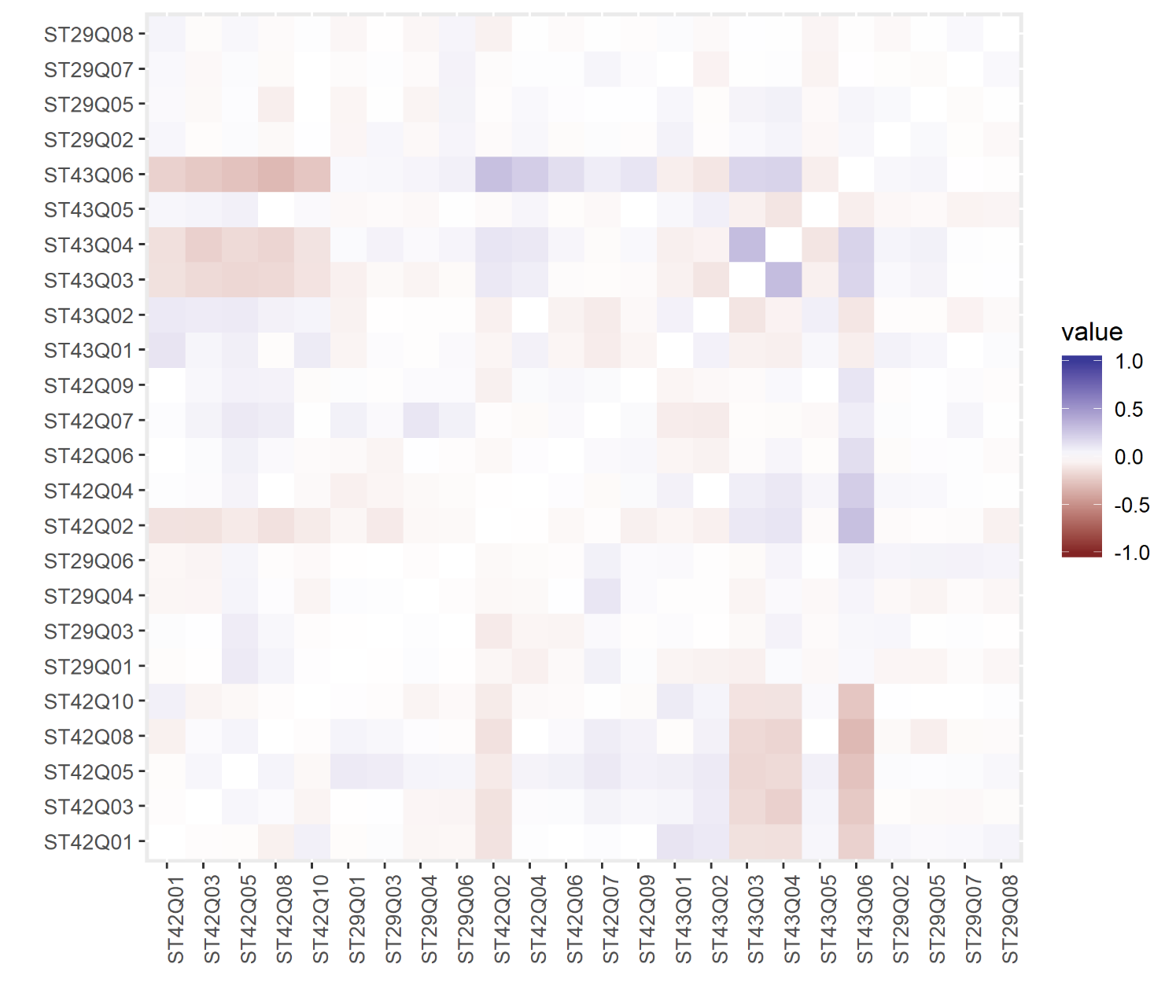
**

**Supplementary Figure 2.** Residual plots of CFA model fit. It plots correlations unexplained by CFA model among 24 response items for MA, MI, MSC, MPC, and INMM. Residual correlations are color coded. Blue means positive correlation and red means negative correlation. ST**Q**, response item names from PISA 2012.


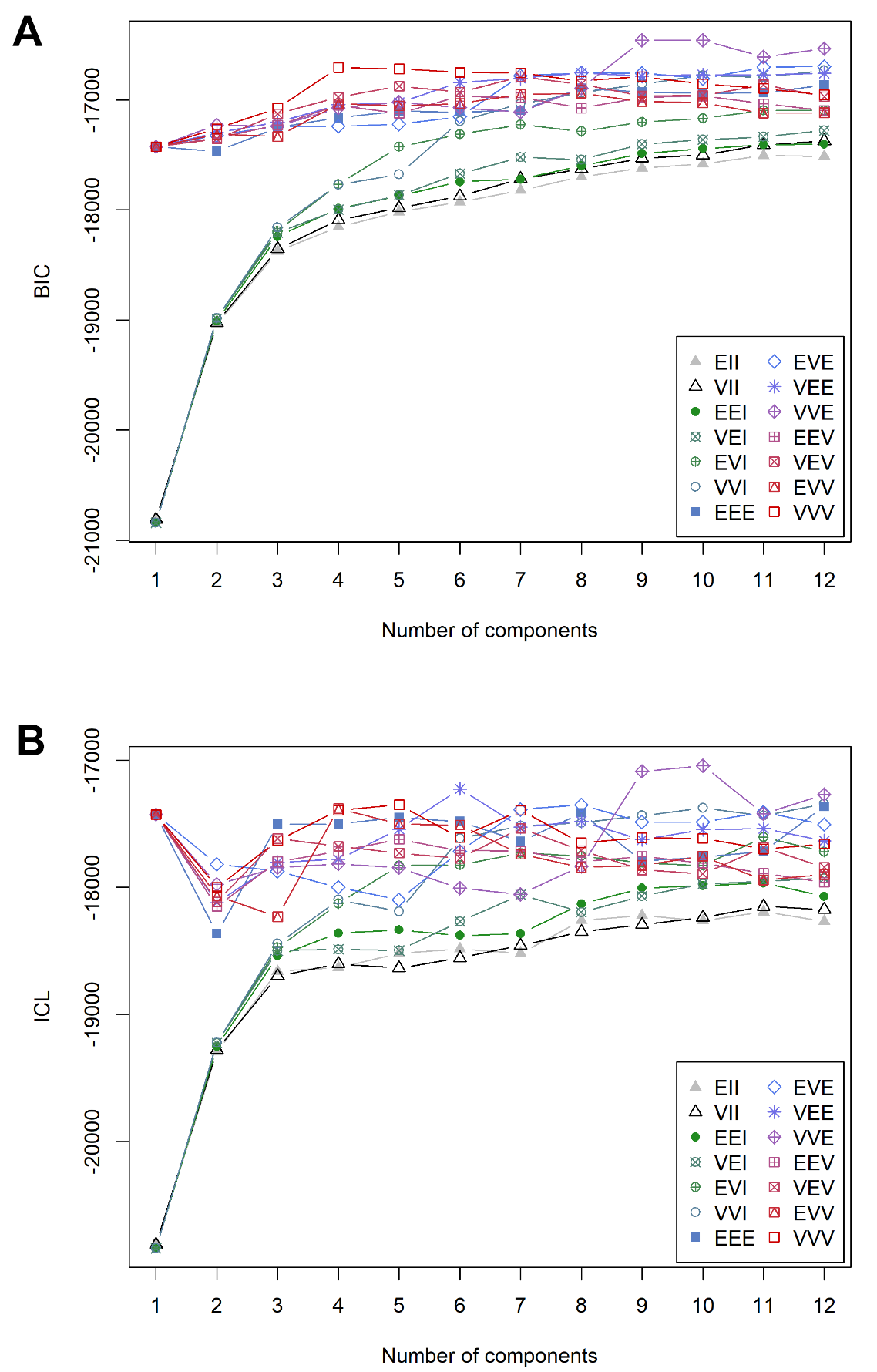


**Supplementary Figure 3.** Selecting model from all 168 models based on BIC **(A)** and ICL **(B)** criteria. The higher value in both BIC and ICL indicates better model. 14 different variance covariance specifications are color and shape coded. X axis indicates number of latent groups. The three letters in model types stand for scale, shape, and orientation of underlying multivariate normal distributions, respectively. E, equal. V, variable. I, identity.


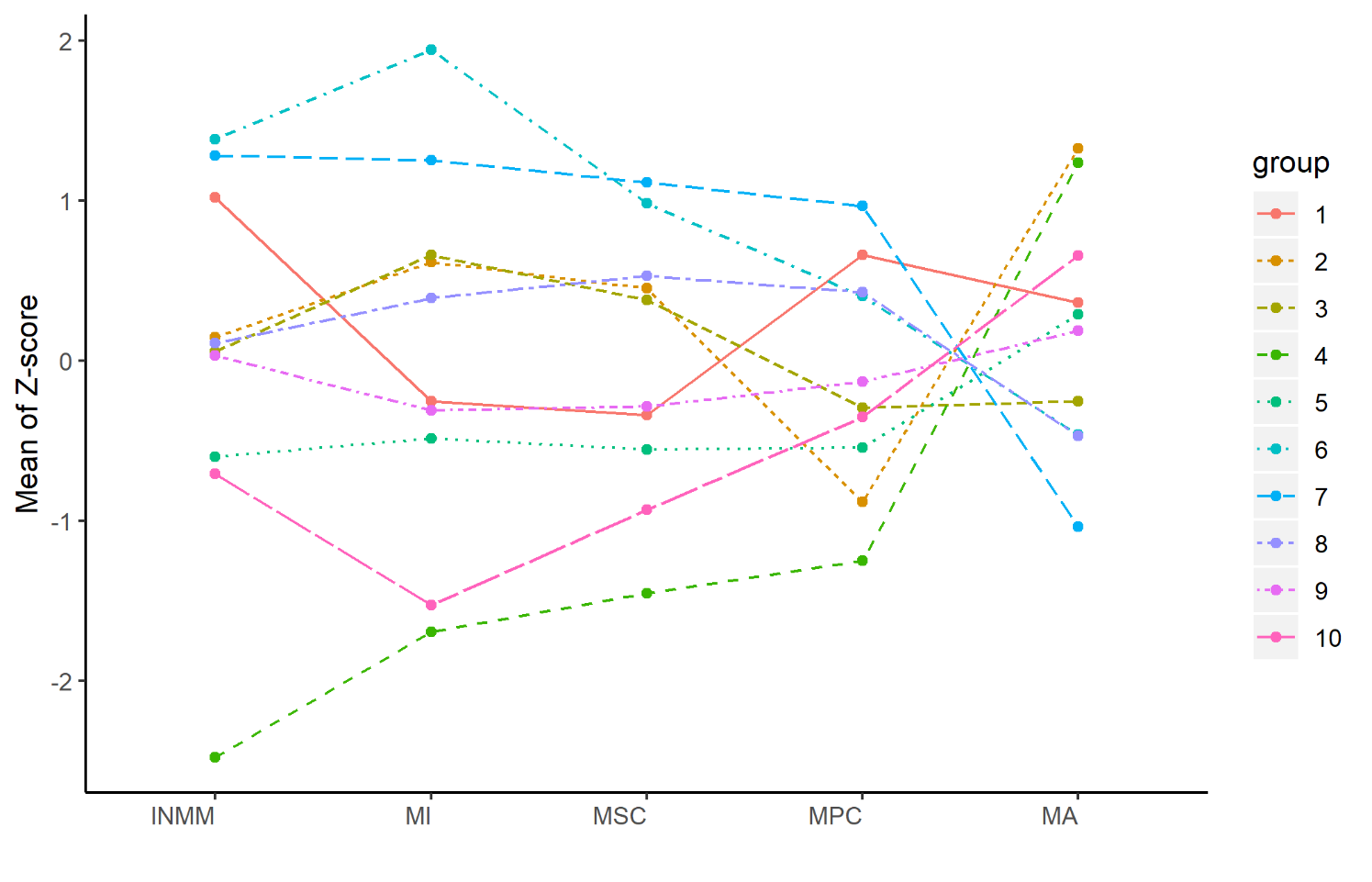


**Supplementary Figure 4.** Latent groups of students from initial LPA. Subgroup means of residualized Z scores of INMM, MI, MSC, MPC, and MA are shown for each latent group. Different subgroups are color coded. MI, interest in math. MSC, math self-concept. MPC, math perceived control. INMM, instrumental motivation of math.


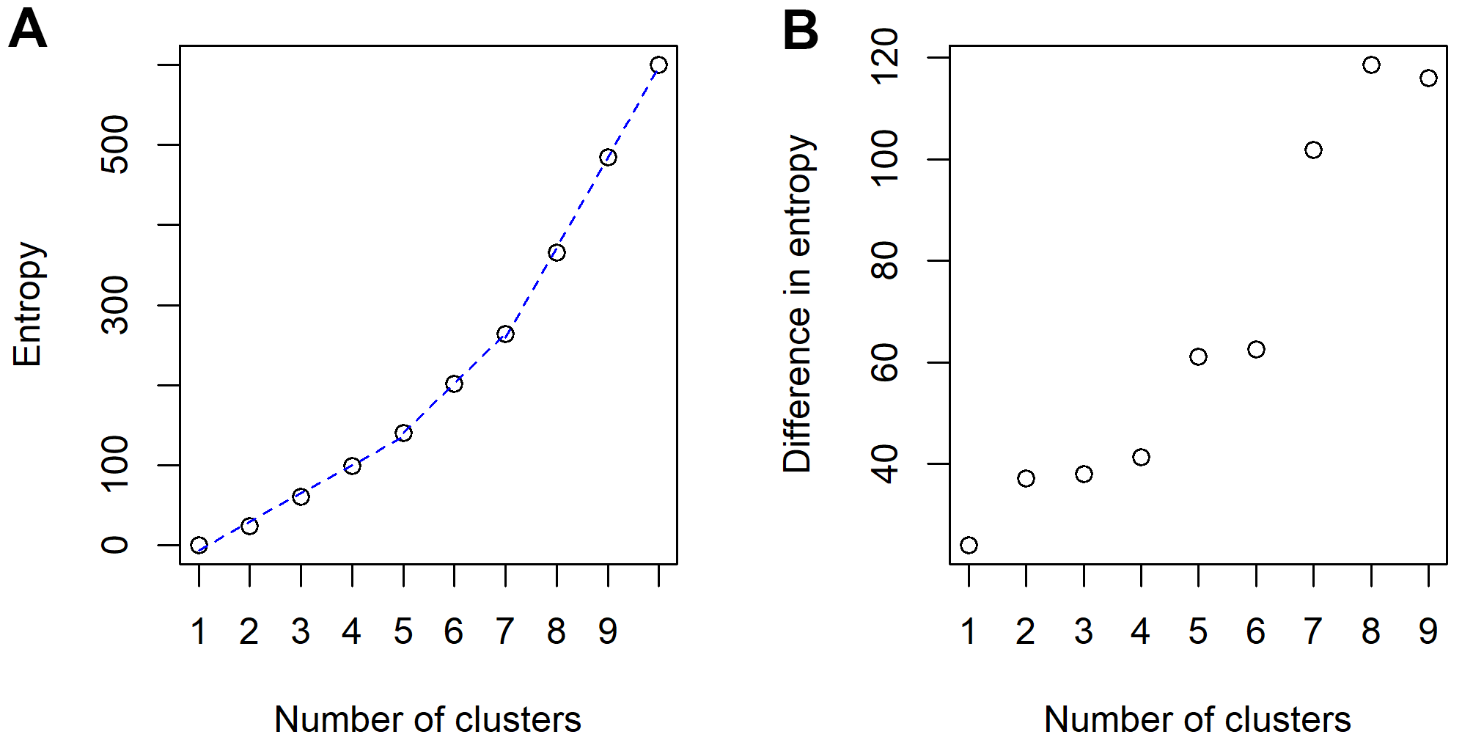


**Supplementary Figure 5.** Secondary clustering of 10 latent groups from LPA. Entropy of merged models **(A)** and decrease of entropy of each merging **(B)** are plotted against number of clusters after merging. Piecewise linear regression lines were plotted as blue dotted line with 3 sections in (A). Two ‘elbow’ points occurred on 5 and 7.

## Supplemental tables

**Supplemental table 1: CFA fitting results**

lavaan 0.6-5 ended normally after 65 iterations

Estimator ML

Optimization method NLMINB

Number of free parameters 58

Number of observations 1464

Model Test User Model:

Test statistic 2352.355

Degrees of freedom 242

P-value (Chi-square) 0.000

Model Test Baseline Model:

Test statistic 23451.398

Degrees of freedom 276

P-value 0.000

User Model versus Baseline Model:

Comparative Fit Index (CFI) 0.909

Tucker-Lewis Index (TLI) 0.896

Loglikelihood and Information Criteria:

Loglikelihood user model (H0) -33211.904

Loglikelihood unrestricted model (H1) -32035.726

Akaike (AIC) 66539.807

Bayesian (BIC) 66846.565

Sample-size adjusted Bayesian (BIC) 66662.317

Root Mean Square Error of Approximation:

RMSEA 0.077

90 Percent confidence interval - lower 0.074

90 Percent confidence interval - upper 0.080

P-value RMSEA <= 0.05 0.000

Standardized Root Mean Square Residual:

SRMR 0.071

Parameter Estimates:

Information Expected

Information saturated (h1) model Structured

Standard errors Standard

Latent Variables:

Estimate Std.Err z-value P(>|z|) Std.lv Std.all

anxiety =~

ST42Q01 1.000 0.656 0.770

ST42Q03 1.077 0.033 32.283 0.000 0.707 0.810

ST42Q05 0.955 0.031 30.729 0.000 0.627 0.776

ST42Q08 0.946 0.032 30.015 0.000 0.621 0.761

ST42Q10 1.135 0.037 30.294 0.000 0.745 0.767

interest =~

ST29Q01 1.000 0.665 0.776

ST29Q03 1.173 0.032 36.843 0.000 0.780 0.870

ST29Q04 1.269 0.033 39.044 0.000 0.844 0.911

ST29Q06 1.201 0.032 37.532 0.000 0.799 0.883

concept =~

ST42Q02 1.000 0.738 0.814

ST42Q04 0.756 0.024 31.557 0.000 0.558 0.740

ST42Q06 1.002 0.026 38.344 0.000 0.740 0.851

ST42Q07 1.148 0.031 36.481 0.000 0.848 0.822

ST42Q09 0.957 0.028 34.736 0.000 0.707 0.794

control =~

ST43Q01 1.000 0.450 0.768

ST43Q02 1.033 0.047 21.825 0.000 0.465 0.636

ST43Q03 0.455 0.054 8.388 0.000 0.205 0.243

ST43Q04 0.533 0.055 9.623 0.000 0.240 0.278

ST43Q05 1.080 0.046 23.415 0.000 0.486 0.688

ST43Q06 0.985 0.057 17.336 0.000 0.444 0.502

instrumot =~

ST29Q02 1.000 0.643 0.815

ST29Q05 1.101 0.028 38.926 0.000 0.708 0.865

ST29Q07 1.206 0.031 38.545 0.000 0.776 0.859

ST29Q08 1.084 0.028 38.260 0.000 0.697 0.855

Covariances:

Estimate Std.Err z-value P(>|z|) Std.lv Std.all

anxiety ~~

interest -0.224 0.015 -14.549 0.000 -0.512 -0.512

concept -0.407 0.021 -19.480 0.000 -0.840 -0.840

control -0.162 0.011 -14.369 0.000 -0.548 -0.548

instrumot -0.158 0.014 -11.595 0.000 -0.375 -0.375

interest ~~

concept 0.350 0.019 18.163 0.000 0.712 0.712

control 0.139 0.011 12.969 0.000 0.463 0.463

instrumot 0.293 0.016 17.812 0.000 0.686 0.686

concept ~~

control 0.212 0.013 16.211 0.000 0.636 0.636

instrumot 0.244 0.016 15.017 0.000 0.514 0.514

control ~~

instrumot 0.166 0.011 15.137 0.000 0.572 0.572

Variances:

Estimate Std.Err z-value P(>|z|) Std.lv Std.all

.ST42Q01 0.295 0.013 22.828 0.000 0.295 0.406

.ST42Q03 0.262 0.012 21.516 0.000 0.262 0.344

.ST42Q05 0.259 0.011 22.668 0.000 0.259 0.398

.ST42Q08 0.281 0.012 23.088 0.000 0.281 0.422

.ST42Q10 0.389 0.017 22.931 0.000 0.389 0.412

.ST29Q01 0.293 0.012 24.198 0.000 0.293 0.398

.ST29Q03 0.196 0.009 21.047 0.000 0.196 0.244

.ST29Q04 0.145 0.008 17.561 0.000 0.145 0.169

.ST29Q06 0.181 0.009 20.206 0.000 0.181 0.221

.ST42Q02 0.278 0.012 22.910 0.000 0.278 0.338

.ST42Q04 0.258 0.011 24.516 0.000 0.258 0.452

.ST42Q06 0.209 0.010 21.473 0.000 0.209 0.276

.ST42Q07 0.345 0.015 22.644 0.000 0.345 0.324

.ST42Q09 0.293 0.012 23.458 0.000 0.293 0.370

.ST43Q01 0.141 0.008 17.869 0.000 0.141 0.410

.ST43Q02 0.318 0.014 22.748 0.000 0.318 0.595

.ST43Q03 0.670 0.025 26.669 0.000 0.670 0.941

.ST43Q04 0.685 0.026 26.536 0.000 0.685 0.922

.ST43Q05 0.263 0.012 21.309 0.000 0.263 0.527

.ST43Q06 0.584 0.023 24.954 0.000 0.584 0.748

.ST29Q02 0.209 0.009 22.276 0.000 0.209 0.335

.ST29Q05 0.168 0.009 19.794 0.000 0.168 0.251

.ST29Q07 0.213 0.011 20.187 0.000 0.213 0.262

.ST29Q08 0.179 0.009 20.460 0.000 0.179 0.269

anxiety 0.431 0.025 16.914 0.000 1.000 1.000

interest 0.443 0.026 17.347 0.000 1.000 1.000

concept 0.545 0.029 18.569 0.000 1.000 1.000

control 0.203 0.013 15.575 0.000 1.000 1.000

instrumot 0.414 0.022 18.536 0.000 1.000 1.000

Schafer, J. (1997). *Analysis of Incomplete Multivariate Data*: Taylor & Francis.
